# Epithelial-derived IL-33 promotes intestinal tumorigenesis in *Apc*^*Min/*+^ mice

**DOI:** 10.1038/s41598-017-05716-z

**Published:** 2017-07-14

**Authors:** Zhengxiang He, Lili Chen, Fabricio O. Souto, Claudia Canasto-Chibuque, Gerold Bongers, Madhura Deshpande, Noam Harpaz, Huaibin M. Ko, Kevin Kelley, Glaucia C. Furtado, Sergio A. Lira

**Affiliations:** 1 Precision Immunology Institute, Icahn School of Medicine at Mount Sinai, New York, 10029 USA; 2 Department of Pathology, Icahn School of Medicine at Mount Sinai, New York, 10029 USA; 3 Department of Developmental and Regenerative Biology, Icahn School of Medicine at Mount Sinai, New York, 10029 USA

## Abstract

Increased expression of Interleukin (IL)-33 has been detected in intestinal samples of patients with ulcerative colitis, a condition associated with increased risk for colon cancer, but its role in the development of colorectal cancer has yet to be fully examined. Here, we investigated the role of epithelial expressed IL-33 during development of intestinal tumors. IL-33 expression was detected in epithelial cells in colorectal cancer specimens and in the *Apc* ^*Min/*+^ mice. To better understand the role of epithelial-derived IL-33 in the intestinal tumorigenesis, we generated transgenic mice expressing IL-33 in intestinal epithelial cells (*V33* mice). *V33 Apc* ^*Min/*+^ mice, resulting from the cross of *V33* with *Apc* ^*Min/*+^ mice, had increased intestinal tumor burden compared with littermate *Apc* ^*Min/*+^ mice. Consistently, *Apc* ^*Min/*+^ mice deficient for IL-33 receptor (ST2), had reduced polyp burden. Mechanistically, overexpression of IL-33 promoted expansion of ST2^+^ regulatory T cells, increased Th2 cytokine milieu, and induced alternatively activated macrophages in the gut. IL-33 promoted marked changes in the expression of antimicrobial peptides, and antibiotic treatment of *V33 Apc* ^*Min/*+^ mice abrogated the tumor promoting-effects of IL-33 in the colon. In conclusion, elevated IL-33 signaling increases tumor development in the *Apc* ^*Min/*+^ mice.

## Introduction

Interleukin (IL)-33 was discovered in 2003 as a nuclear factor expressed by high endothelial venules^1^ and subsequently described as a pro-inflammatory cytokine belonging to the IL-1 cytokine family^2^. IL-33 is primarily expressed in non-hematopoietic cells, including fibroblasts, epithelial cells and endothelial cells, and by cells of hematopoietic origin, particularly in macrophages and dendritic cells (DCs)^2^. IL-33 is a dual-function protein, functioning as conventional cytokine via its extracellular form (mature-IL-33) or as a transcriptional regulator via its nuclear form (full length-IL-33). The former is mediated by its binding to the heterodimeric receptor complex consisting of the ST2 receptor (ST2L) and the widely expressed IL-1R accessory protein^3, 4^. ST2 is widely expressed on immune cells including eosinophils, basophils, macrophages, T helper 2 cells (Th2 cells), regulatory T cells, NK cells, B cells and group 2 innate lymphoid cells (ILC2)^5–7^. The broad distribution of the ST2 receptor in immune cells suggests the involvement of IL-33 in the pathogenesis of a wide range of diseases^8^, including asthma, rheumatoid, arthritis and atherosclerosis.

Several studies document upregulation of IL-33 in the inflamed colonic mucosa of patients with inflammatory bowel disease (IBD)^9–14^. Recent findings suggest that enterocyte-derived IL-33 is induced and maintained by inflammatory mediators, because IL-33 is detected in the nuclei of epithelial cells of colonic crypts in acute ulcerative colitis, but undetectable during remission^15^. It is known that IBD patients have an increased risk for development of colitis-associated colorectal cancer (CAC), but the role of IL-33 in the development of colorectal cancer has yet to be fully examined.

Colorectal cancer (CRC) is the second most prevalent cause of death from cancer in the Western world^16^. The majority of CRC are sporadic, arising from dysplastic adenomatous polyps. The etiology of human CRC has been linked to genetic variables, such as adenomatous polyposis coli (APC) proteins, inflammatory processes, and gut microbiota. A multi-step process leads to the accumulation of genetic alterations that confer a selective growth advantage to the colonic epithelial cells and drive the transformation from normal epithelium to adenomatous polyp and finally to invasive colorectal cancer^17^. Loss of heterozygosity for APC in intestinal epithelial cells (IECs) activates Wnt signaling through stabilization of β-catenin, which is sufficient to initiate polyp formation. The *Apc* ^*Min/*+^ mice, an animal model of human familial adenomatous polyposis^18^, carry a mutation in this tumor suppressor gene and have a predisposition to multiple intestinal neoplasia (Min)^19^.

Recent evidence suggests that IL-33 can function as a novel epithelial “alarmin”^20^, because it is released as a danger signal by damaged, stressed, or necrotic cells to alert the immune system of a local threat. Indeed, increased expression of full-length IL-33 is detected in ulcerative colitis epithelium^10, 11, 13^. However, the role of IL-33 in intestinal inflammation is incompletely understood, with both pro- and anti- inflammatory effects being reported^21–24^. Similarly, the role of IL-33/ST2 signaling in intestinal tumorigenesis is also unclear. Using IL-33 deficient mice, Maywald *et al*.^25^ have shown that IL-33 derived from the tumor epithelium promotes polyposis through the coordinated activation of stromal cells in *Apc* ^*Min/*+^ mice. Ablation of IL-33/ST2 signaling by using ST2 deficient mice significantly prevents tumor formation in the azoxymethane (AOM)/dextran sodium sulphate (DSS) model of CRC^26^. These results are in contrast to those by Malik *et al*.^27^ who have found that IL-33 deletion increases tumor incidence in the AOM/DSS model.

Here we report that IL-33 is expressed by epithelial cells within human and mouse intestinal tumors. To study the relevance of increased IL-33 expression to tumor development we generated IL-33 transgenic mice and mice deficient for its receptor (ST2). Increased expression of IL-33 in the gut epithelium of *Apc* ^*Min/*+^ mice promoted intestinal tumorigenesis, with expansion of ST2^+^ regulatory T cells and induction of a Th2 cytokine milieu in the colon. The increased tumorigenesis elicited by IL-33 was independent of additional activation of the β-catenin pathway, but appeared to be dependent on ST2 and the microbiota. IL-33 promoted marked changes in the expression of antimicrobial peptides and antibiotic treatment of *V33 Apc* ^*Min/*+^ mice abrogated the tumor promoting-effects of IL-33 in the colon.

## Results

### Elevated expression of IL-33 in tumor epithelial cells of CRC patients and *Apc*^*Min/*+^ mice

**Figure 1:**
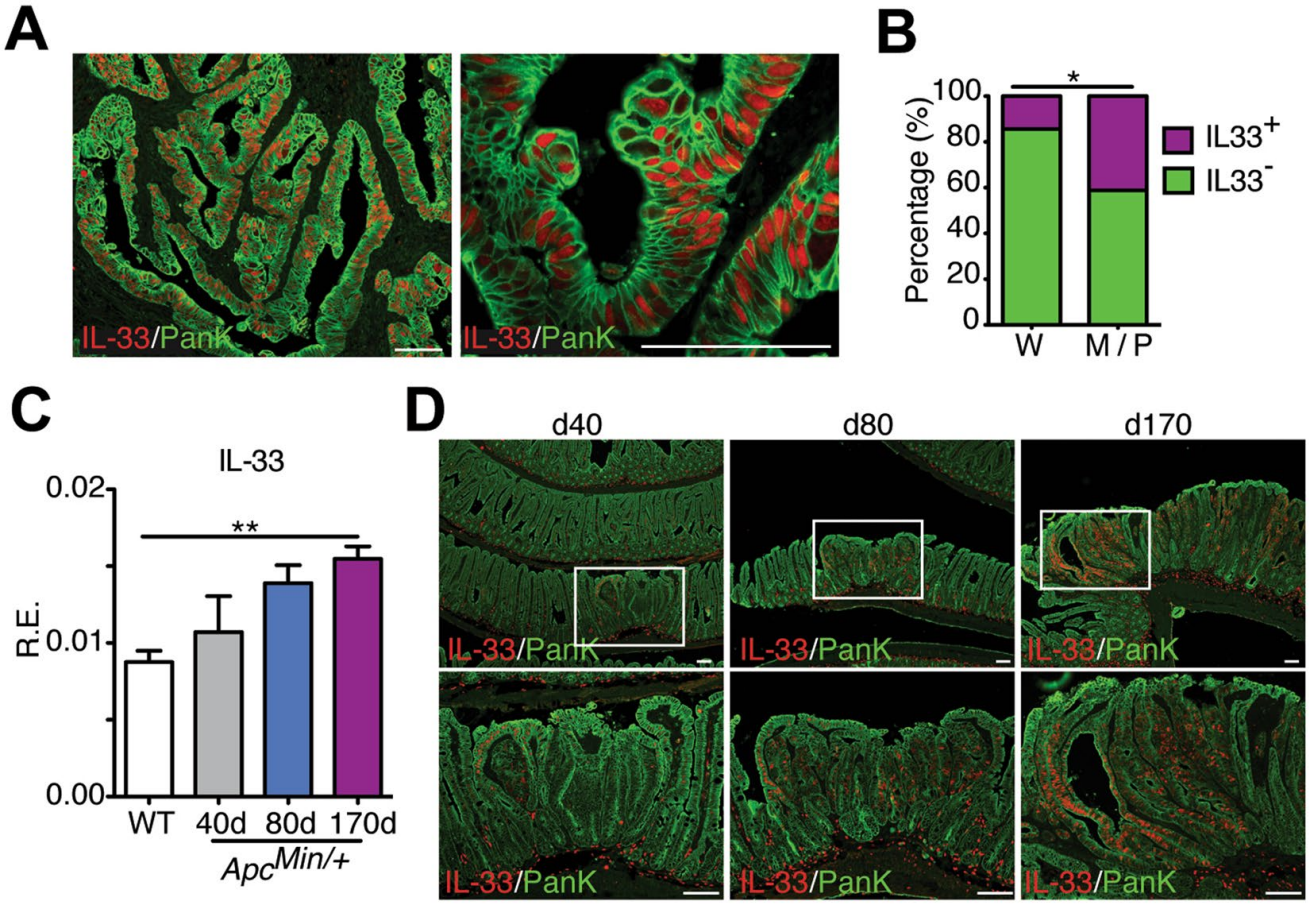
IL-33 expression in human colorectal cancer patients and in *Apc* ^*Min/*+^ mice model. (**A**) Paraffin sections from a tissue microarray of human colorectal cancer (CRC) patients were stained with IL-33 (red) and Pan keratin (green). Representative photographs show expression of IL-33 by intestinal epithelial cells (IECs) in poorly differentiated adenocarcinomas. Scale bars, 100 μm. (**B**) The percentage of IL-33 immunolabeling in the IECs from 21 well-differentiated CRC patients (W) and 68 moderate- and poor-differentiated CRC patients (M/P). *P < 0.05, Fisher exact test. (**C**) Relative expression levels of IL-33 mRNA were analyzed by qPCR in the gut of *Apc* ^*Min/*+^ mice at various ages. Data were normalized to the expression levels of the Ubiquitin transcript. Means ± s.e.m., n = 4 per group. **P < 0.01, nonparametric Mann-Whitney test. (**D**) Immunofluorescence staining for IL-33 (red) and Pan keratin (green) in *Apc* ^*Min/*+^ mice at different ages. Zoomed-in boxed area shows IL-33 immunolabeling in the IECs in *Apc* ^*Min/*+^ mice. Scale bars, 50 μm.

Given that IL-33 expression is significantly up-regulated in UC^10, 11^, we asked if expression of IL-33 was increased in UC-associated tumors. Analysis of a tumor tissue microarray of UC-CRC patients (n = 47) obtained from our MSSM Biorepository showed that there was expression of IL-33 in the epithelium in 9 out of 47 samples (19.2%). We also found epithelial expression of IL-33 in 15 out of 42 non-IBD CRC tumors (35.7%). Taken together, our data indicate that IL-33 is expressed by tumor epithelial cells in approximately 30% of all samples (n = 89). IL-33 immunostaining was detected mostly in epithelial cells and localized to the nucleus (Fig. 1A). Of note, we found expression of IL-33 in approximately 15% of well-differentiated CRC cells (n = 21), and in almost 40% of moderate- and poorly-differentiated CRC cells (n = 68) (Fig. 1B). Therefore, higher expression of IL-33 in tumor epithelial cells was observed in moderate- and poorly-differentiated CRC patients.

To further investigate a link between tumorigenesis and IL-33 expression we examined IL-33 expression in *Apc* ^*Min/*+^ mice. We found that the expression of IL-33 mRNA was significantly increased throughout tumor development (Fig. 1C). Immunostaining experiments showed that IL-33 was expressed by epithelial cell in adenomatous areas, compared with adjacent normal tissue (Fig. 1D). IL-33 was detected in epithelial cells within microadenomas by d40. Tumor progression results in the expansion of microadenomas and an increase in tumor size. With time, the number of epithelial cells expressing IL-33 increased significantly (Fig. 1D). Together, these results document expression of IL-33 in intestinal tumors of humans and mice and suggest that IL-33 is involved in their development.

### Generation of transgenic mice expressing IL-33 in the intestinal epithelium

**Figure 2:**
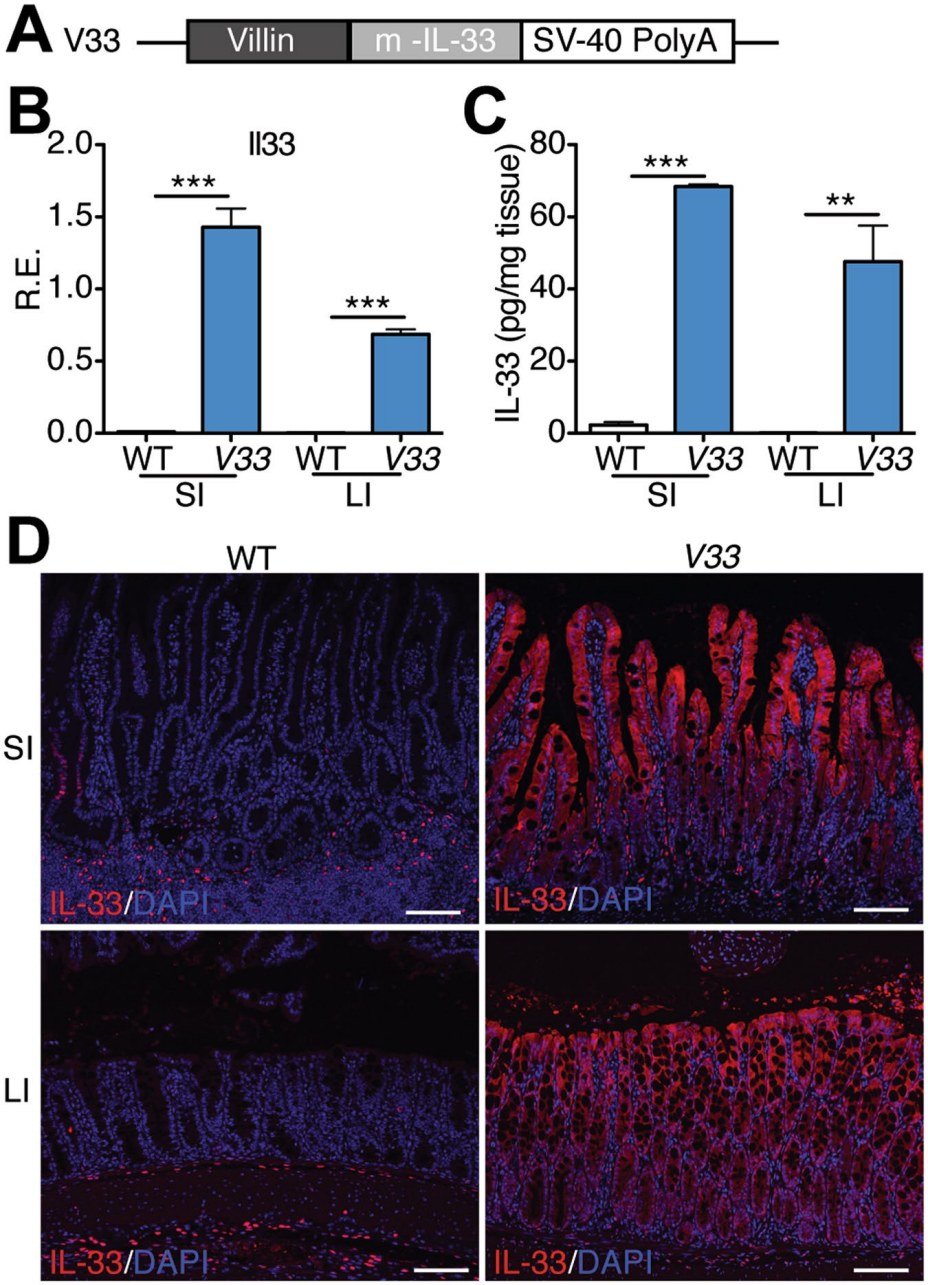
Generation of transgenic mice expressing IL-33 in the gut epithelium. (**A**) Scheme for generation of *V33* mice. A transgene encoding IL-33 mature form (m-IL-33) under the control of the murine villin promoter (9 kb) was used to generate *V33* mice. (**B**) Relative expression levels of transgenic IL-33 mRNA were analyzed by qPCR in the small intestine (SI) and large intestine (LI) of wild-type (WT) and *V33* mice. Data were normalized to the expression levels of the Ubiquitin transcript. Means ± s.e.m., n = 6 per group. ***P < 0.001, one-way ANOVA. (**C**) Enzyme linked immunosorbent assay of IL-33 in the gut explants from WT and *V33* mice. Data were normalized to the weight of the intestine explant. Means ± s.e.m., n = 4 per group. **P < 0.01, ***P < 0.001, one-way ANOVA. (**D**) Immunofluorescence staining for IL-33 (red) in the gut of WT and *V33* mice. Cell nuclei were counterstained with DAPI (blue). Notice that transgenic expression of IL-33 in the cytoplasm of intestinal epithelial cells in *V33* mice. Scale bars, 50 μm.

Next we examined if epithelial-cell derived IL-33 contributes to tumor development. IL-33 is a dual-function protein, functioning as a conventional cytokine and/or as a transcriptional regulator. To reduce the complexity and focus on the cytokine function of IL-33, we cloned the cDNA encoding the mature form of IL-33 (amino acids 95–267, lacking the nuclear localization signal) downstream of the villin promoter^28^. The transgene was injected into mouse eggs and 4 transgenic lines were derived from founder mice. These animals are referred to as *V33* mice (Fig. 2A). The *V33* mice were healthy, reproduced normally, and did not develop intestinal tumors. To examine IL-33 expression in the intestine of these transgenic mice we performed qPCR in RNA extracted form the small and large intestine. As expected, we detected increased expression of IL-33 mRNA in the small intestine and large intestine in the *V33* transgenic mice compared with their littermate control wild-type (WT) mice (Fig. 2B). To examine expression of IL-33 protein, we performed enzyme linked immunosorbent assay in the gut extracts and found that IL-33 was elevated in the intestine of transgenic mice compared to WT mice (Fig. 2C). Finally, we examined the cellular expression of IL-33. Because we expressed the mature form of IL-33, we expected that it should be located in the cytoplasm rather than the nucleus. Immunostaining of intestinal sections showed that IL-33 immunoreactivity was indeed detected in the cytoplasm of transgenic, but not control intestinal epithelial cells (Fig. 2D). In sum, we confirmed appropriate tissue and cellular expression of the *V33* transgene.

### IL-33/ST2 signaling promotes polyp growth in *Apc*^*Min/*+^ mice

**Figure 3:**
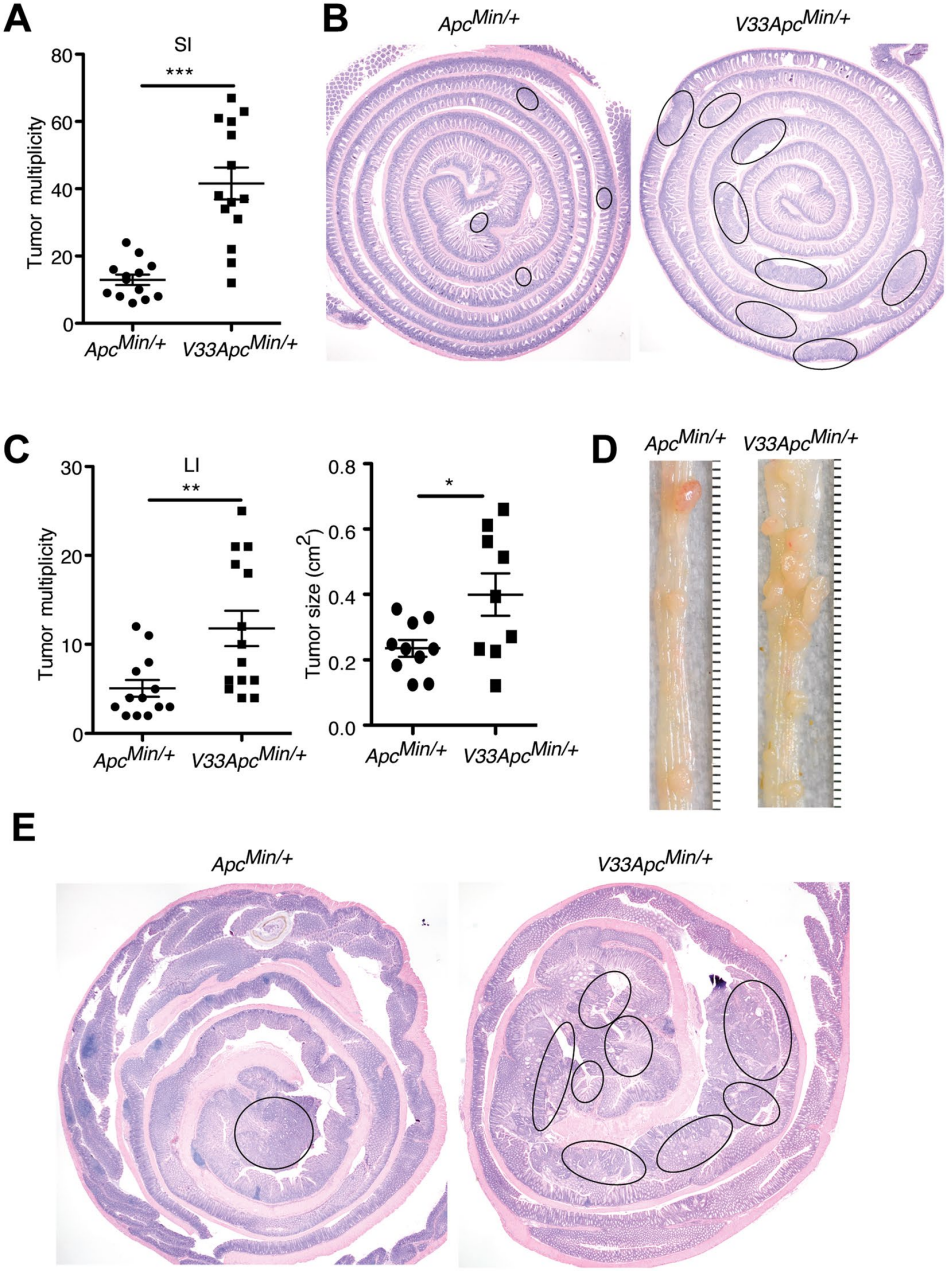
IL-33 promotes intestinal polyp growth in *Apc* ^*Min/*+^ mice. (**A**) *V33 Apc* ^*Min/*+^ mice carried a significantly higher number of polyps in the SI than *Apc* ^*Min/*+^ mice at d120. (**B**) Representative hematoxylin and eosin (H&E) staining of ileum of *Apc* ^*Min/*+^ mice and *V33 Apc* ^*Min/*+^ mice at d120. Individual tumors are highlighted by black lines. (**C**) Tumor number (left) and tumor size (right) of LI in *Apc* ^*Min/*+^ mice and *V33 Apc* ^*Min/*+^ mice at d120. (**D**) Representative gross specimens from the indicated genotype analyzed at d120. (**E**) Representative H&E staining of LI of *Apc* ^*Min/*+^ mice and *V33* *Apc* ^*Min/*+^ mice at d120. Individual tumors are highlighted by black lines. Graphs show means ± s.e.m. *P < 0.05, **P < 0.01, ***P < 0.001, nonparametric Mann-Whitney test.

To explore the potential impact of epithelial-specific IL-33 on IEC tumors, we crossed *V33* mice with *Apc* ^*Min/*+^ mice to generate *V33 Apc* ^*Min/*+^ mice. Next we measured the number of tumors (polyps) in *V33 Apc* ^*Min/*+^ mice and their littermate control *Apc* ^*Min/*+^ mice at 120 days of age. We found that *V33 Apc* ^*Min/*+^ mice had more polyps in the small intestine than *Apc* ^*Min/*+^ mice (Fig. 3A), which was confirmed by histological analysis (Fig. 3B). Because the Villin promoter drives transgene expression in the entire intestinal epithelium (Fig. 2D), we also investigated the effect of tumor burden of *V33 Apc* ^*Min/*+^ mice in the large intestine. Importantly, we found a higher mean number and increased mean size of colonic polyps in *V33 Apc* ^*Min/*+^ mice compared to their *Apc* ^*Min/*+^ littermates (Fig. 3C,D). Histological analysis confirmed increased tumor burden, and increased tumor size in the large intestine of *V33 Apc* ^*Min/*+^ mice (Fig. 3E).

**Figure 4:**
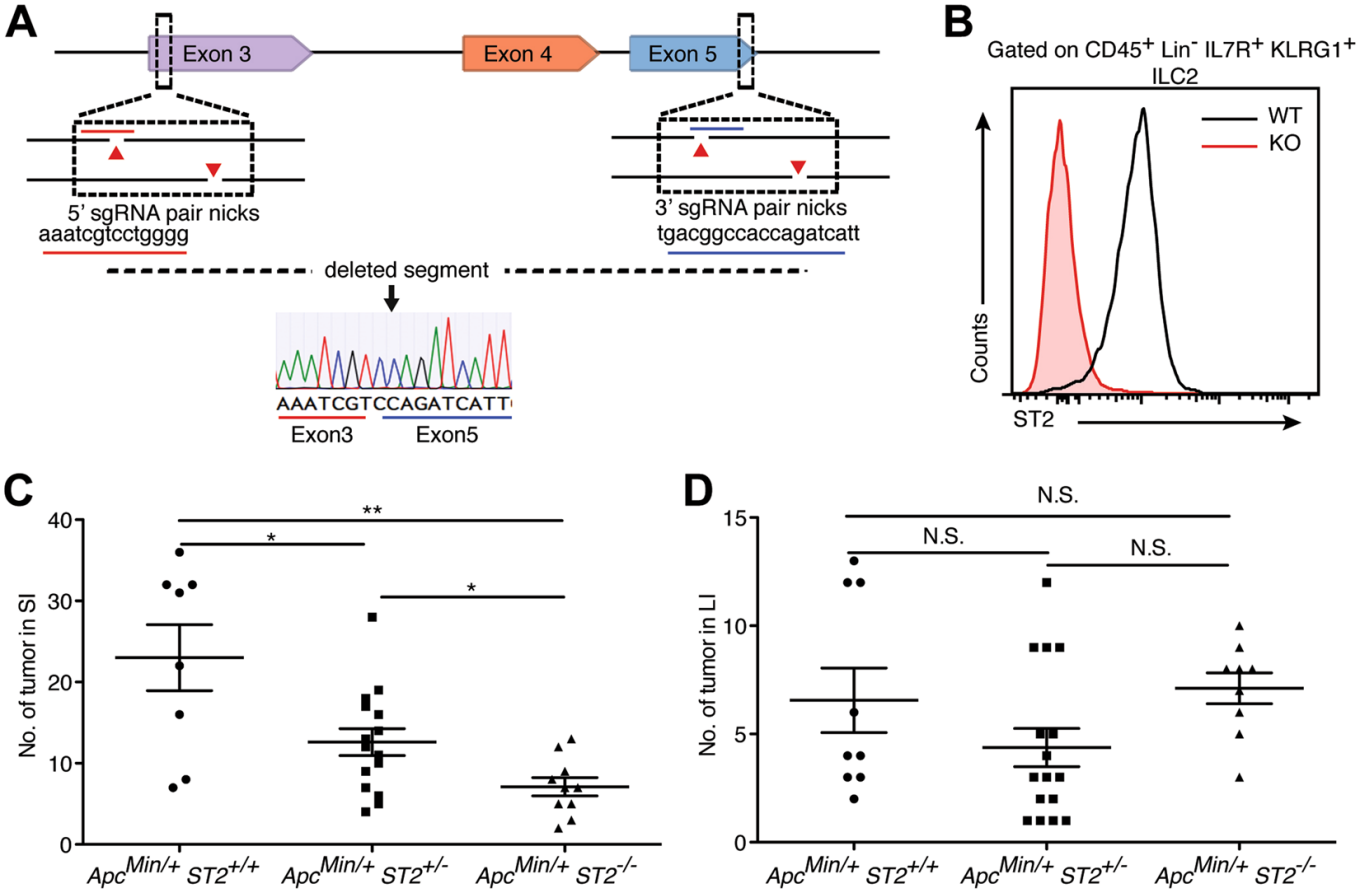
Genetic ablation of ST2 reduces polyp burden in *Apc* ^*Min/*+^ mice. (**A**) Scheme for generation of *ST2* ^−/−^ mice by CRISPR/Cas9n technology. The two g-RNA targeting sequences are underlined in red and blue. Sequencing confirmed the deletion of exons 4 and 5. (**B**) Flow cytometric analysis of the expression of ST2 on ILC2 (CD45^+^ Lin^−^ IL7R^+^ KLRG1^+^) isolated from the mesenteric fat of WT and *ST2* ^−/−^ mice. Notice the absence of ST2 expression in the ILC2 isolated from *ST2* ^−/−^ mice. (**C**,**D**) Quantification of the number of tumors in the small (**C**) and large (**D**) intestine of *Apc* ^*Min/*+^ *ST2* ^+/+^, *Apc* ^*Min/*+^ *ST2* ^+/−^ and *Apc* ^*Min/*+^ *ST2* ^−/−^ mice. Graphs show means ± s.e.m. *P < 0.05, **P < 0.01, N.S. means not significant, nonparametric Mann-Whitney test.

We next tested whether IL-33/ST2 signaling deficiency affected the development of tumors. To do so, we first generated ST2-deficient mice using the CRISPR/Cas9n technology^29^. Three ST2-deficient lines were derived from founder mice. In these animals, the exons 4 and 5 of the ST2 gene, which correspond to the IL-33 binding site^30^, were deleted (Fig. 4A). To test if the genetic changes induced functional inactivation of ST2, we examined ST2 expression in ILC2 of ST2^−/−^ mice. Flow cytometric analysis showed that ST2 expression was completely abrogated in ILC2 of the ST2^−/−^ mice, verifying that the engineered mutation is a null allele (Fig. 4B). We then crossed *ST2* ^−/−^ mice with *Apc* ^*Min/*+^ mice to produce *Apc* ^*Min/*+^ deficient for ST2 (*Apc* ^*Min/*+^ *ST2* ^−/−^) mice and littermate controls (*Apc* ^*Min/*+^ *ST2* ^+/−^, *Apc* ^*Min/*+^ *ST2* ^+/+^). Gross analysis of the intestine of these animals at 120 d of age indicated a reduction in the number of polyps in the small intestine of *Apc* ^*Min/*+^ *ST2* ^−/−^ mice compared with *Apc* ^*Min/*+^ *ST2* ^+/−^ and *Apc* ^*Min/*+^ *ST2* ^+/+^ mice (Fig. 4C). However, there was no effect on the tumor development in the large intestine in these sets of experiments (Fig. 4D).

Taken together these results confirm a positive role for IL-33 signaling in tumor development in the *Apc* ^*Min/*+^ background.

### IL-33 increases colonic ST2^+^ regulatory T cells

IL-33 influences various immune cells during differentiation, immune responses, and homeostasis. Its receptor ST2 is widely expressed on immune cells including eosinophils, basophils, macrophages, Th2 cells, regulatory T cells, NK cells, and ILC2^5–7^. To start examining possible cellular differences in our mice, we analyzed these cell populations. We found that there were no differences in the relative and absolute number of colonic eosinophils, macrophages, monocytes, DC, neutrophils, NK cells, NKT cells, B cells (Supplementary Figure 1) and ILC2 (Supplementary Figure 2) between the *V33 Apc* ^*Min/*+^ mice and *Apc* ^*Min/*+^ mice. However, there was an increase in the CD3^+^ population in *V33 Apc* ^*Min/*+^ mice compared to *Apc* ^*Min/*+^ mice (Supplementary Figure 1).

**Figure 5:**
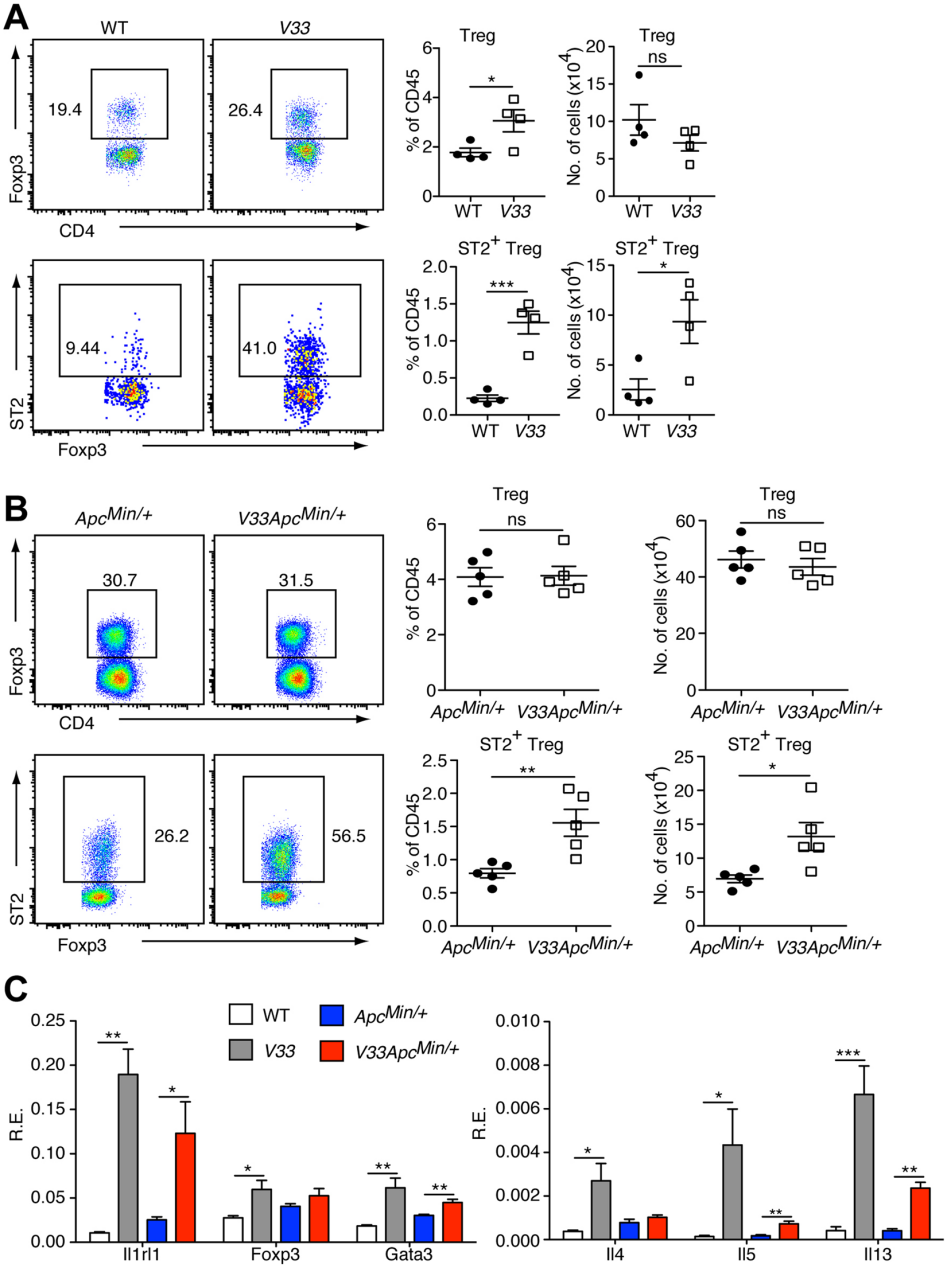
Epithelial-derived IL-33 signaling expands colonic ST2^+^ regulatory T (Treg) cells and induces a Th2 cytokine profile. (**A**) Relative and absolute number of Treg cells (CD4^+^Foxp3^+^) in the colon of *Apc* ^*Min/*+^ mice and *V33 Apc* ^*Min/*+^ mice at d120. Left, representative flow cytometry plots gated on CD45^+^ cells; right, statistical data show means ± s.e.m. (**B**) Relative and absolute number of ST2^+^ Treg cells in the colon of *Apc* ^*Min/*+^ mice and *V33 Apc* ^*Min/*+^ mice at d120. Left, representative flow cytometry plots gated on CD45^+^ CD4^+^ cells; right, statistical data show means ± s.e.m. (**C**) Relative expression levels of ST2^+^Treg and Th2 signature genes were analyzed by qPCR in sorted T cells of colonic lamina propria of WT, *V33*, *Apc* ^*Min/*+^ mice and *V33 Apc* ^*Min/*+^ mice at d120. Graphs show means ± s.e.m., n = 4–5/group. *P < 0.05, **P < 0.01, ***P < 0.001, one-way ANOVA.

Next we examined whether expression of IL-33 affected the number of Treg, especially ST2^+^ Treg in the intestine of *V33* mice. We found that expression of IL-33 in the epithelium led to an increase the relative number of colonic Treg cells (Fig. 5A). Importantly, *V33* mice also had more colonic ST2^+^ Treg cells compared with WT control in both relative and absolute number (Fig. 5A). Because *V33 Apc* ^*Min/*+^ mice had higher tumor burden than *Apc* ^*Min/*+^ mice, we asked whether promotion of intestinal tumorigenesis was correlated with increased number of ST2^+^ Treg cells in the gut. Flow-cytometric analysis demonstrated that there was no difference in the frequency and total number of colonic Treg cells between the *V33 Apc* ^*Min/*+^ mice and *Apc* ^*Min/*+^ mice (Fig. 5B). However, *V33 Apc* ^*Min/*+^ mice had more colonic ST2^+^ Treg cells than *Apc* ^*Min/*+^ mice (relative and absolute number) (Fig. 5B). Since epithelium-expressed IL-33 promoted tumor growth not only in the LI (Fig. 3C) but also in the SI (Fig. 3A), we also investigated the ST2^+^ Treg cells in the SI. First, we analyzed ST2^+^ Treg cells between *Apc* ^*Min/*+^ mice and WT mice. We found that *Apc* ^*Min/*+^ mice had more Treg cells (relative and absolute number) in the SI than WT controls (Supplementary Figure 3A and B). Importantly, *Apc* ^*Min/*+^ mice had more ST2^+^ Treg cells in the SI than WT mice (Supplementary Figure 3C and D). Similarly, *V33 Apc* ^*Min/*+^ mice had more ST2^+^ Treg cells in the SI than *Apc* ^*Min/*+^ mice (Supplementary Figure 3E). Therefore, we conclude that IL-33 increases the numbers of intestinal ST2^+^ Treg cells in both small and large intestine.

To better evaluate the phenotype of colonic lamina propria T cells, we next sorted colonic T cells (CD45^+^ CD3^+^ cells) from mice of all genotypes and performed qPCR. In line with flow cytometric results, mRNA levels of ST2 and Foxp3 were upregulated in colonic T cells of *V33* mice compared with WT mice (Fig. 5C). Of note, IL-4, IL-5, IL-13 and Gata3 mRNAs were also upregulated in colonic T cells of *V33* mice (Fig. 5C), suggesting that *V33* colonic T cells showed a Th2-predominant phenotype. Accordingly, qPCR analysis of sorted T cells also confirmed increased expression of ST2, Foxp3 and Th2 cytokines in *V33 Apc* ^*Min/*+^ mice when compared with *Apc* ^*Min/*+^ mice (Fig. 5C). Together these results demonstrate that epithelial-derived IL-33 induces expansion of ST2^+^ Treg and promotes a Th2-predominant cytokine milieu.

### IL-33 induces colonic alternatively activated macrophages in *Apc*^*Min/*+^ mice

**Figure 6:**
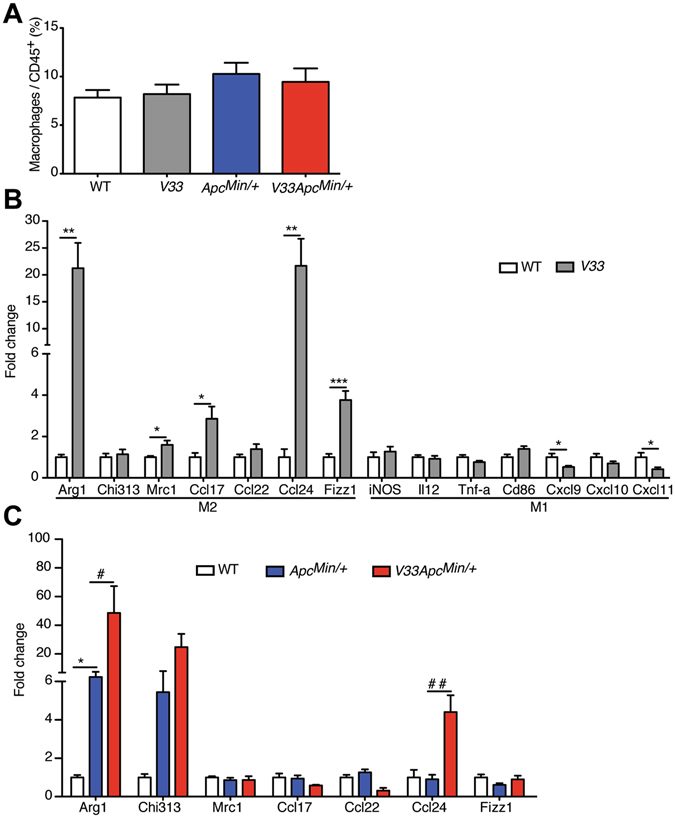
IL-33 regulates alternative activation of colonic macrophages. (**A**) Relative number of macrophages (CD45^+^CD11b^+^F480^+^) in the colon of WT, *V33*, *Apc* ^*Min/*+^ mice and *V33 Apc* ^*Min/*+^ mice at d120. Graphs show means ± s.e.m., n = 3–5/group. (**B**) Relative expression levels of M1 and M2 macrophage signature genes were analyzed by qPCR in sorted macrophages of colonic lamina propria of WT mice and *V33* mice at d120. (**C**) Relative expression levels of M2 macrophage signature genes were analyzed by qPCR in sorted macrophages of colonic lamina propria of WT mice, *Apc* ^*Min/*+^ mice and *V33 Apc* ^*Min/*+^ mice at d120. Data were normalized to fold change from age-matched WT littermate control mice. Graphs show means ± s.e.m., n = 3–5/group. *P < 0.05, **P < 0.01, ***P < 0.001, versus WT group. ^#^ *P* < 0.05, ^##^ *P* < 0.01, versus *Apc* ^*Min/*+^ group.

Macrophages are a major component of the leukocyte infiltrates in various tumor stroma. ST2 is constitutively expressed at the surface of mouse macrophages and IL-33 has been implicated in their activation^31, 32^. IL-33 promotes or amplifies the expression of chemokines by M1 or M2-polarized macrophages^33^. We first determined if overexpression of IL-33 could change the relative number of macrophages in the colon. Flow-cytometric results demonstrated that there was no difference in the relative number of colonic macrophages among the WT, *V33*, *Apc* ^*Min/*+^ mice and *V33 Apc* ^*Min/*+^ mice (Fig. 6A), indicating that epithelium-derived IL-33 did not affect the number of colonic macrophages. To determine whether epithelium-derived IL-33 had a role in induction of colonic macrophages, we investigated mRNA expression of representative M1 and M2 genes on the sorted macrophages (CD45^+^CD11b^+^F4/80^+^ cells) from the colonic lamina propria leucocytes (LPL) in WT, *V33*, *Apc* ^*Min/*+^ and *V33 Apc* ^*Min/*+^ mice. qPCR analysis data, normalized to fold change from age-matched WT littermate control mice, showed that expression of the M2 markers arginase 1 (*Arg*-*1*), *Mrc*-*1*, *Fizz1*, *Ccl17*, *Ccl22* and *Ccl24*, was higher in colonic macrophages of *V33* mice than in those of WT mice (Fig. 6B). Expression of the M1 markers *iNOS*, *IL*-*12p35*, *Tnf*-*a* and *Cd86* was not different between *V33* mice and WT mice, but expression of *Cxcl9*, *Cxcl10* and *Cxcl11*, key chemokines produced by M1 macrophages, was lower in the colonic macrophages of *V33* mice compared with WT mice (Fig. 6B). Thus, overexpression of IL-33 in the gut activated colonic macrophages to M2 phenotypes. Of note, we also found increased expression of *Arg*-*1*, *Chi313* and *Ccl24* in the colonic macrophages of *V33 Apc* ^*Min/*+^ mice when compared with WT mice (Fig. 6C). Similarly, when compared with *Apc* ^*Min/*+^ mice, *V33 Apc* ^*Min/*+^ mice had colonic macrophages with increased expression of M2 markers (Fig. 6C). Taken together, the results suggest that IL-33 induces colonic alternatively activated macrophages in *Apc* ^*Min/*+^ mice.

### IL-33 promotes tumorigenesis independent of the β-catenin pathway in IEC

Loss of APC protein leads to a deregulated WNT/β-catenin pathway^34^, as APC is part of the β-catenin destruction complex^18, 34^. Therefore, β-catenin plays a central role in intestinal proliferation and neoplastic transformation. To determine whether activation of the Wnt pathway plays a role in the increased polyp burden observed in *V33 Apc* ^*Min/*+^ mice, we performed β-catenin immunostaining within size-matched tumors from *V33 Apc* ^*Min/*+^ mice and *Apc* ^*Min/*+^ mice. Polyps from both genotypes showed an equivalent increase in cytoplasmic and nuclear accumulation of β-catenin and frequencies of cells with nuclear β-catenin (an indicator of activated β-catenin) (Supplementary Figure 4A). To further validate these results we examined expression of the Wnt target genes in the IEC isolated from tumor areas and adjacent normal area in *V33 Apc* ^*Min/*+^ mice and *Apc* ^*Min/*+^ mice. The mRNA levels of *c*-*myc*, *Birc5*, *Cd44*, *Lgr5*, *Tcf7*, *Mmp7*, and *Ephb3* were significantly increased in tumor area compared with non-tumor area (Supplementary Figure 4B). However, there was no difference in the expression of these genes when we compared tumors from *V33 Apc* ^*Min/*+^ mice and *Apc* ^*Min/*+^ mice (Supplementary Figure 4B). These results suggest that IL-33 does not affect β-catenin nuclear translocation in dysplastic epithelium.

### IL-33 changes antimicrobial gene expression in the epithelium

Next, we examined if epithelium expressed IL-33 affected IEC function. Injection of IL-33 has been shown to impair epithelial barrier function^13^. We therefore examined epithelial permeability in *V33* mice. There were no significant difference in intestinal permeability in WT and *V33* mice assessed by measuring serum FITC-Dextran levels 5 h after administration (Supplementary Figure 5).

**Figure 7:**
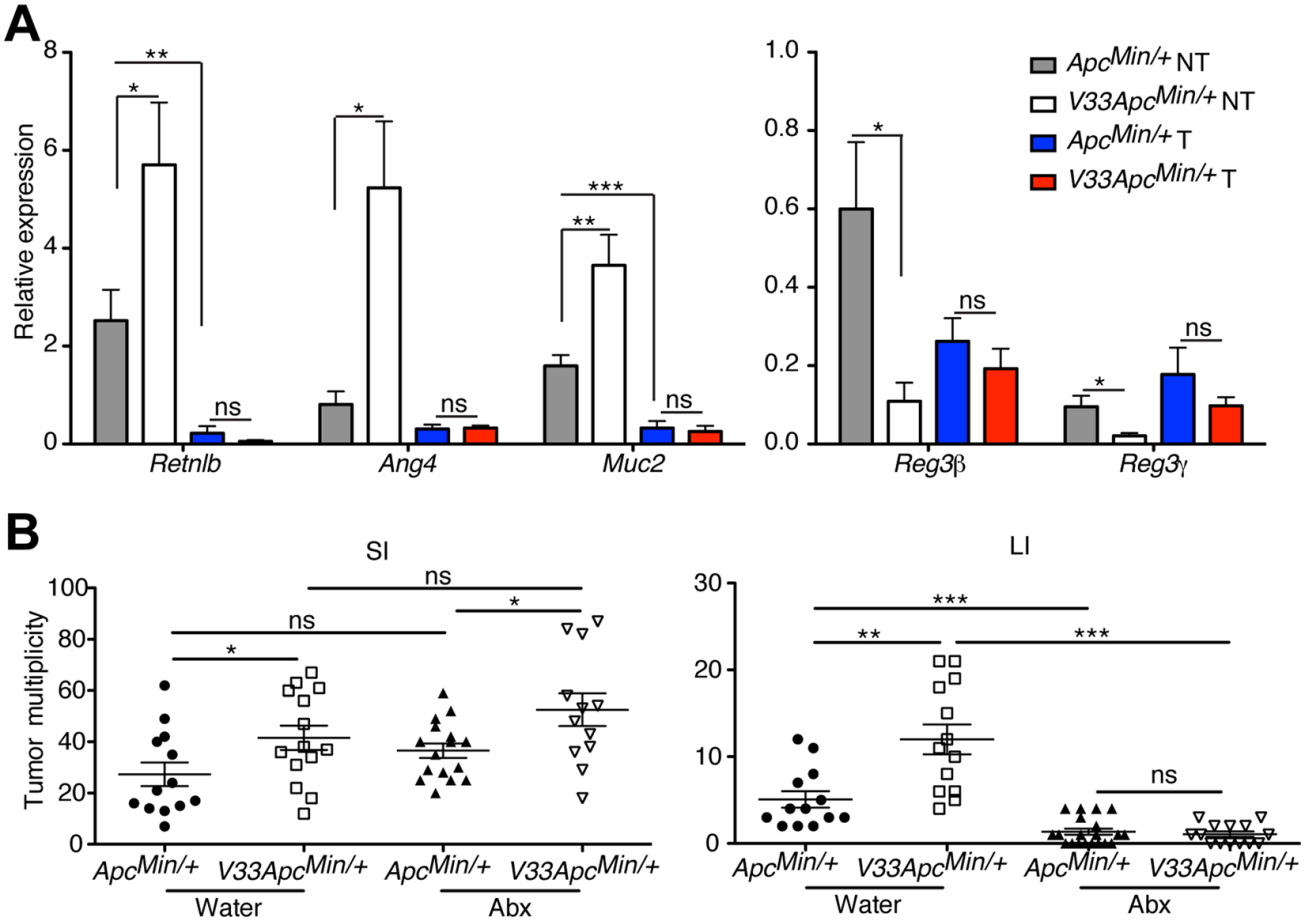
Epithelial-derived IL-33 alters antimicrobial genes expression in the colon. (**A**) Relative expression levels of *Reg3b*, *Reg3g*, *Ang4*, *Retnlb* and *Muc2* were analyzed by qPCR in the colon of size-matched tumor area (T) and adjacent non-tumor normal area (NT) of *Apc* ^*Min/*+^ mice and *V33 Apc* ^*Min/*+^ mice at d120. Data were normalized to the expression levels of the Ubiquitin transcript. Graphs show means ± s.e.m. n = 4–5/group. *P < 0.05, **P < 0.01, ***P < 0.001, one-way ANOVA. (**B**) Number of polyps in the intestine of *Apc* ^*Min/*+^ mice and *V33 Apc* ^*Min/*+^ mice receiving water or antibiotic continuously from weaning to 120 days post-birth. Graphs show means ± s.e.m. *P < 0.05, **P < 0.01, ***P < 0.001, nonparametric Mann-Whitney test.

Antimicrobial peptides produced by epithelial cells protect the host intestinal mucosa against microorganisms^35^. Since IL-33 is expressed by epithelial cells in adenomatous areas compared with adjacent normal tissue in *Apc* ^*Min/*+^ mice (Fig. 1D), we decided to examine whether IL-33 affected antimicrobial gene expression. We found that cells within the adenomas of *Apc* ^*Min/*+^ mice expressed less *Retnlb* and *Muc2* mRNA (Fig. 7A) than cells in normal adjacent tissue. Furthermore, compared with *Apc* ^*Min/*+^ mice, *V33 Apc* ^*Min/*+^ mice expressed less *Reg3b* and *Reg3g* in the colonic mucosa, and more *Retnlb*, *Ang4* and *Muc2* mRNA (Fig. 7A). These changes, however, were not observed when comparing tumor areas from both genotypes (Fig. 7A).

### Pro-tumorigenic activities of IL-33 are partially dependent on the microbiota in the colon

Antimicrobial factors are critical in controlling the microbiota, which in turn affects CRC development. To examine a potential contribution of the microbiota to development of tumors in our model system, we treated *V33 Apc* ^*Min/*+^ mice and *Apc* ^*Min/*+^ mice with antibiotics (ampicillin metronidazole, neomycin, vancomycin)^28, 36^. Antibiotics were provided in the drinking water continuously, from weaning to 120 days post-birth. Antibiotic treatment significantly decreased polyp load in the colon of *Apc* ^*Min/*+^ mice and *V33 Apc* ^*Min/*+^ mice, but did not affect tumorigenesis in the small intestine (Fig. 7B). Consistent with previous report^37^, colonic polyps are highly sensitive to antibiotic treatment rather than small intestine. Importantly, after antibiotic treatment, there was no difference in the number of tumors between *Apc* ^*Min/*+^ mice and *V33 Apc* ^*Min/*+^ mice (Fig. 7B). These observations suggest that the protumorigenic activities of IL-33 at least in the large intestine may be partly mediated by changes in the microbiota.

## Discussion

We show here that expression of IL-33 is increased within epithelial cells of intestinal tumors in humans and mice and that constitutive expression of IL-33 in the context of the *Apc* mutation leads to increased tumorigenesis. Together these results strongly suggest that IL-33 is a factor involved in intestinal tumorigenesis.

Inflammation is a risk factor for CRC development. Increased expression of IL-33 has been detected in intestinal samples of patients with ulcerative colitis^9–14^. A recent study shows that IL-33 is present in the nuclei of enterocytes in scattered colonic crypts in acute ulcerative colitis, but is not present in these cells at remission^15^. Genetic studies have suggested a role for the *Il33* gene in the risk of developing IBD^38^. However, the precise effect of epithelial-derived IL-33 to the gut inflammatory conditions has remained unclear. Experimental data obtained using different animal models of intestinal inflammation have produced conflicting results^21–24^, with IL-33 having both pro- and anti- inflammatory effects. Increased expression of IL-33 is detected in SAMP mice, a spontaneous model of chronic intestinal inflammation characterized by a mixed Th1/Th2 immune phenotype. In this model, IL-33 was shown to potently increase the production of the pro-inflammatory cytokines, such as IL-5, IL-6 and IL-17^11^. IL-33 appears to have a protective effect in trinitrobenzene sulfonic acid-induced colitis, mostly driven by a Th1 immune response^39^. In addition, IL-33 signaling protects from murine oxazolone colitis by supporting intestinal epithelial function^40^. Disparate results also exist regarding the role of IL-33 in the DSS-induced colitis model. Following epithelial barrier disruption caused by DSS administration, IL-33 injection worsened colitis, inducing the recruitment of neutrophils to the site of inflammation^41–43^ and induction of type 2 cytokines^44–46^, whereas, during the recovery phase, it showed a prominent effect in promoting mucosal healing^41, 43^ and inducing goblet cell proliferation^42^, eventually restoring epithelial barrier function. IL-33 has also been reported to increase expression of the growth factor amphiregulin to enhance colonic mucin responses in DSS-induced colitis model^47^. In the present study, we used the villin promoter to drive IL-33 expression in mice, which targeted IL-33 expression specifically to the gut epithelium. Expression of IL-33 in this setting did not cause significant inflammation (data not shown). Therefore, we conclude that epithelial-derived IL-33 promoted intestinal tumorigenesis in *Apc* ^*min/*+^ mice independent of its pro-inflammatory properties.

As discussed above, the role of IL-33 in tumor biology is still controversial. Some studies show that IL-33 inhibits tumor growth by stimulating production of IFN-γ by CD8^+^ T cells and Th1 cells^48, 49^. Systemic over-expression of IL-33 in transgenic mice promotes NK and CD8^+^ T cell function and inhibits tumor growth and metastasis^50^. Overexpression of IL-33 in tumor cells strongly inhibited tumor growth by increasing the numbers of tumor-infiltrating NK cells and CD8^+^ T cells, and their production of IFN-γ^51^. However, other studies report that IL-33 promotes tumor growth. Jovanovic *et al*.^52^, using a breast cancer model, have shown that IL-33 promotes cancer progression through increased intratumoral accumulation of immunosuppressive cells and by diminishing innate antitumor immunity.

In our study, we examined expression of IL-33 in humans and in mice. We show here that the expression of IL-33 is higher in the epithelium of moderate- and poorly-differentiated CRC than in well-differentiated tumors suggesting a positive correlation of IL-33 expression in the epithelium and tumor grade. Our data are consistent with an earlier report that IL-33/ST2 pathway contributes to human colorectal cancer^53^. Our study provides evidence for dynamic changes in IL-33 expression in polyps of *Apc* ^*Min/*+^ mice. Epithelial expression of mature IL-33 correlated positively with tumor growth, and deletion of ST2 decreased tumor incidence. Since CRC associated IL-33 is predominantly nuclear, we also generated transgenic mice that expressed full-length IL-33 in the epithelium to test its role on the tumor development. Overexpression of full-length IL-33 in the context of the *Apc* mutation also led to increased tumorigenesis (data not shown), which is consistent with the phenotype driven by epithelial expression of mature IL-33.

IL-33 has been shown to affect various immune cells during the inflammation and cancer development. Here we demonstrate that two main targets of IL-33 (T cells and macrophages) are affected *in vivo*. We demonstrate changes in the number and function of T cells. More specifically, we document changes in the number of ST2^+^ Tregs. This effect is likely to be caused by expansion of ST2^+^ Tregs, because IL-33 does not appear to promote their differentiation from ST2^-^ Treg^54^. Tregs from IL-33-treated mice display enhanced capacities to suppress IFN-γ production by effector T cells^55^, and thus contribute to their pro-tumorigenic activities in *Apc* ^*Min/*+^ mice. In addition, our results show increased expression of IL-4, IL-5, IL-13, Foxp3 and Gata3 in colonic T cells of *V33* mice (Fig. 5). ST2^+^ Tregs exhibit a Th2-biased character, express GATA-3 and produce the Th2 cytokines IL-5 and IL-13 *in vitro* ^56^. Expression of IL-33 in our model did not affect the numbers of other IL-33 target cells such as eosinophils, monocytes, DC, neutrophils, NK cells, NKT cells, B cells and ILC2 cells (Supplementary Figure 1 and Supplementary Figure 2). Whether IL-33 modifies their function to promote tumorigenesis requires additional evaluation.

The ability of macrophages to affect tumor development is well described in the literature^57^. Two general mechanisms could be invoked to explain how IL-33 could affect macrophage function in our model. First, IL-33 could affect the macrophage phenotype. It is well documented that IL-33 induces macrophages to switch from the M1 to the M2 phenotype *in vivo* ^58, 59^. In agreement with these results, we found in our study that IL-33 expression was associated with polarized M2 phenotype. Mechanistically this effect could be due to Th2 cytokines, since it has been demonstrated that Th2 cytokines are drivers of M2 polarization^58^. We suggest that the increased expression of Th2-type cytokines by ST2^+^ Tregs in our model may be critical for the development of the M2 phenotype. Second, IL-33 could promote expression of protumorigenic factors such as prostaglandin E2, as recently reported^60^.

In addition to immune cells, we also find that IL-33 may act on epithelial cells to promote tumor growth. Intestinal epithelial cells express ST2 in both mice and humans^13^. We found that expression of IL-33 in the gut epithelium increases *Ang4*, *Retnlb* and *Muc2* transcription levels. We suggest that IL-33 promotes the colonic goblet cells to secrete these factors. IL-33 induces goblet cell hyperplasia, and stimulates mucous production at mucosal surfaces^2, 42^. Ang4 is an antimicrobial peptide important in epithelial host defence in the small intestine^61^, and is produced by goblet cells in the large intestine^62^. The expression of Retnlb is tightly restricted to intestinal goblet cells, from where it is secreted apically into the intestinal lumen^63^. Th2 cytokines can induce expression of Retnlb by goblet cells^64^. Reduced Retnlb transcriptional levels are associated with fewer IL-13 mRNA transcripts in the intestines of IL-33^−/−^ compared with WT mice^42, 65^. MUC2, a major goblet cell mucin, has been used as the goblet cell marker. Reg3β and Reg3γ are expressed by different epithelial cell types in colon including enterocytes and goblet cells. Absence of Muc2 in *Muc2* ^−/−^ mice results in up-regulation of Reg3β and Reg3γ expression, suggesting altered bacterial-epithelial signaling and an innate defense response^66^. Although it is unknown whether IL-33 affects enterocytes, we observed that expression of IL-33 in the epithelium increased Muc2 expression together with decreased the Reg3β and Reg3γ expression in the colon.

Changes in gut microbiota have been associated with tumor growth^67^. The growing tumors can selectively alter the microbial community causing expansion of pathogenic bacteria that promote tumor development^37^. Immune system components can also regulate the host microbiota that modulate the susceptibility to tumor^27^. As shown here, epithelium expressed IL-33 changes antimicrobial gene expression in the epithelium. Abnormal expression of these antimicrobial genes could potentially modify the microbiota and promote dysbiosis, and favor tumor development^68^. In addition, the changes in the immune parameters induced by IL-33 expression (increase in ST2^+^ Tregs, induction of Th2 cytokines and M2 macrophages) could also contribute to regulation of the commensal bacteria that modulate the susceptibility to tumor. To test if the microbiota could potentially affect IL-33 induced tumorigenesis, we treated mice with a cocktail of broad-spectrum antibiotics that produces a profound shift in the composition and abundance of intestinal microbiota in the murine intestine^36^. Our results show that *V33 Apc* ^*Min/*+^ mice have more polyps in the small intestine and large intestine than *Apc* ^*Min/*+^ mice. However, after antibiotic treatment, there was no difference in the number of colonic tumors between *Apc* ^*Min/*+^ mice and *V33 Apc* ^*Min/*+^ mice, suggesting that some of the protumorigenic activities of IL-33 in the colon may be mediated by changes in the microbiota. This effect could be mediated via direct regulation of antimicrobial gene expression, or by other factors associated with immune system. Indeed, a recent study by Malik *et al*.^27^ suggest that IL-33 protects against colitis and CRC by regulating intestinal IgA production, which in turn affects the composition of the microbiota.

In summary, we show here that expression of IL-33 is increased within epithelial cells of intestinal tumors in humans and mice; that IL-33 promotes tumor development when overexpressed in intestinal cells of *Apc* ^*Min/*+^ mice; and that deletion of IL-33 signaling by deletion of ST2, significantly reduces tumorigenesis in these animals. IL-33 overexpression did not increase Wnt signaling but promoted expansion of ST2^+^ Treg cells and induction of colonic alternatively activated macrophages. IL-33 also promoted marked changes in the expression of antimicrobial peptides and antibiotic treatment of *V33 Apc* ^*Min/*+^ mice abrogated the tumor promoting-effects of IL-33 in the colon. Therefore, the results presented here, reveal a previously unappreciated ability of epithelial expressed-IL-33 to control tumor progression in the intestine through effects on both immune and epithelial cells (Supplementary Figure 6).

## Materials and Methods

### Ethics approval

All animal experiments in this study were approved by the Institutional Animal Care and Use Committee of Icahn School of Medicine at Mount Sinai, and were performed in accordance with the approved guidelines for animal experimentation at the Icahn School of Medicine at Mount Sinai (IACUC-2015-0004).

### Mice

C57BL/6 mice and *Apc* ^*Min/*+^ mice were purchased from The Jackson laboratory (Bar Harbor, ME). Mice were maintained under specific pathogen-free conditions.

### Generation of transgenic mice expressing IL-33 in the intestinal epithelium

The cDNA of IL-33 mature form (95–267 amino acids) was cloned into a pBS-Villin vector that contained a 9 kb segment of the mouse villin promoter^69^. The pBS-Villin/m-IL-33 plasmid was verified by sequencing, and the transgene was isolated from the plasmid by restriction enzyme digestion and gel purification. To generate transgenic mice, the transgene was microinjected into C57BL/6 mouse eggs^28^. Identification of the transgenic *V33* mice was done by PCR amplification using the following primers: 5′- ggctgtgatagcacacagga-3′ and 5′- gttccttggatgctcaatgtgt -3′.

### Generation of ST2-deficient mice

We used the CRISPR/Cas9n system to delete exons 4 and 5 of the ST2^30^. To do so we designed two pairs of sgRNA flanking these exons, as described before^29^. Plasmids expressing each sgRNA were prepared by ligating oligos into *Bbs*I site of pX460 (http://www.addgene.org/48873/)^29^. To construct a single plasmid for injection, four PCR-amplified sgRNA driven by U6 promoter were inserted into the plasmid (pX460) containing hCas9n. All these reagents were validated by sequencing. C57BL/6 eggs were injected with the pX460 plasmid containing 4 sgRNAs and hCas9n at 1 ng/ul as described previously^70^. Identification of mice carrying the deletion of exons 4 and 5 was done by PCR amplification using the primers: 5′- cccatgtatttgacagttacgg-3′ and 5′- tggcttttatatccaggaactca-3′.

### Tissue microarray of human colorectal cancer patients

A colorectal cancer tissue microarray was generated as previously described^71^. Briefly, sections were obtained from de-identified paraffin blocks and HE stained. A certified pathologist defined representative tumor regions. Small tissue cylinders with a diameter of 0.6 mm were taken from selected areas of each donor block using a tissue chip micro-arrayer (Beecher Instruments, Silver Spring, MD, USA) and transferred to a recipient paraffin block. The recipient paraffin block was cut in 2 μm paraffin sections using standard techniques. The use of the pathologic samples was approved and reviewed by the Ethics Committee of the Icahn School of Medicine at Mount Sinai.

### Antibodies

For flow cytometry, the following fluorochrome-conjugated anti-mouse antibodies were used: CD45 (30-F11), CD11b (M1/70), CD3e (145-2C11), CD4 (GK1.5), F4/80 (BM8), Foxp3 (FJK-16s), CD127 (A7R34), KLRG1 (2F1) were from eBioscience; IL-33R-biotin (T1/S2, clone DJ8) from MD Bioproducts; Streptavidin PE-Cyanine7 was from eBioscience; FITC anti-mouse Lineage Cocktail with Isotype Ctrl was from Biolegend. For immunofluorescence staining, purified anti-mouse IL-33 polyclonal antibody (AF3626) and anti-human IL-33 polyclonal antibody (AF3625) were from R&D Systems; beta-Catenin antibody (Cat: #9587) was from Cell Signaling Technology and Pan-keratin antibody (PCK-26) was from Abcam. Alexa Fluor 488-conjugated Donkey antibody to mouse IgG (A21202), Alexa Fluor 594-conjugated Donkey antibody to goat IgG (A11058) and Alexa Fluor 594-conjugated goat antibody to rabbit IgG (A11037) were from Invitrogen.

### Flow cytometry and sorting

Cell suspensions from the lamina propria were prepared as described previously^36^. All cells were first pre-incubated with anti-mouse CD16/CD32 for blockade of Fc γ receptors, then were washed and incubated for 40 min with the appropriate monoclonal antibody conjugates in a total volume of 200 μl PBS containing 2 mM EDTA and 2% (vol/vol) bovine serum. DAPI (Invitrogen) was used to distinguish live cells from dead cells during cell analysis and sorting. For detection of intracellular Foxp3, a Foxp3 Staining Buffer Set (eBioscience) was used for fixation and permeabilization of the cells. Stained cells were analyzed on a LSRII machine using Diva program (BD Bioscience) or purified with a MoFlo Astrios cell sorter (DakoCytomation). Cells were >98% pure after sorting. Data were analyzed with FlowJo software (TreeStar).

### Enzyme-linked immunosorbent assay

Small pieces of small intestine or colon (~5 mm of mid-part) were isolated, rinsed in HBSS/BSA, weighed, and cultured overnight in 12-well tissue culture plates (Costar) in 1000 μl complete DMEM at 37 °C in an atmosphere containing 5% CO_2_. After centrifugation to pellet debris, culture supernatants were transferred to fresh tubes and stored at −80 °C. IL-33 was quantified in the supernatant of intestinal explant cultures from *V33* and WT mice by enzyme-linked immunosorbent assay (ELISA) according to standard manufacturer’s recommendations (eBioscience) and the results were normalized to the weight of the intestinal explant.

### Reverse-transcription polymerase chain reaction

Total RNA from tissues/sorted cells was extracted using the RNeasy mini/micro Kit (Qiagen) according to the manufacturer’s instructions. Complementary DNA (cDNA) was generated with Superscript III (Invitrogen). Quantitative PCR was performed using SYBR Green Dye (Roche) on the 7500 Real Time System (Applied Biosystems) machine. Thermal cycling conditions used were as follows: 50 °C for 2 min and 95 °C for 10 min, 40 cycles of 95 °C for 15 s, 60 °C for 1 min, followed by dissociation stage. Results were normalized to the housekeeping gene Ubiquitin. Relative expression levels were calculated as 2^(Ct(Ubiquitin)-Ct(gene))^. Primers were designed using primer express 2.0 software (System Applied Biosystems).

### Histology and immunofluorescence staining

Tissues were dissected, fixed in 10% phosphate-buffered formalin, and then processed for paraffin sections. Five-micrometer sections were stained with hematoxylin and eosin for histological analyses. For immunofluorescence staining, five-micrometer sections were dewaxed by immersion in xylene (twice for 5 minutes each time) and hydrated by serial immersion in 100%, 90%, 80%, and 70% ethanol and PBS. Antigen retrieval was performed by microwaving sections for 20 minutes in Target Retrieval Solution (DAKO). Sections were washed with PBS (twice for 10 minutes each time), and blocking buffer (10% BSA in TBS) was added for 1 hour. Sections were incubated with primary antibody in blocking buffer overnight at 4 °C. After washing, conjugated secondary Abs were added and then incubated for 35 min. Cell nuclei were stained using DAPI. The slides were next washed and mounted with Fluoromount-G (Southern Biotech). Images were captured using a Nikon fluorescence microscope. Colocalization was performed with ImageJ (http://rsbweb.nih.gov/ij/) and the colocalization finder plug-in.

### FITC-Dextran assay

Mice were kept without food and water for 4 hr and then FITC-dextran (#FD4-1G, Sigma) was administered by oral gavage at a concentration of 40 mg/ml in phosphate-buffered saline (PBS) in 400 μl (16 mg) per mouse (~800 mg/kg). Five hours later, plasma was collected from peripheral blood, then mixed 1:1 with PBS and analyzed on a plate reader at an excitation wavelength of 485 nm and an emission wavelength of 535 nm.

### Antibiotic treatment

For reduction of the intestinal microbiota, a ‘cocktail’ of antibiotics containing ampicillin 1 g/L, metronidazole 1 g/L, neomycin 1 g/L, and vancomycin 0.5 g/L (Sigma Aldrich) in the drinking water was provided to the mice through their drinking water continuously from weaning to 120 days post-birth. Antibiotic treatment was renewed every week.

### Statistics

Differences between groups were analyzed with Student’s t tests or nonparametric Mann-Whitney test. For the comparison of more than two groups a one-way ANOVA followed by a Bonferroni multiple comparison test was performed. All statistical analyses were performed with GraphPad Prism 5 software.

## Electronic supplementary material

Supplementary Figures
