# Supplementary material for: Epithelial-derived IL-33 promotes intestinal tumorigenesis in ApcMin/+ mice
Source: Sci Rep. 2017 Jul 14;7:5520. doi: 10.1038/s41598-017-05716-z (PMC5511216; doi:10.1038/s41598-017-05716-z)
Supplement: Supplementary file 1 — Supplementary Figures [file 41598_2017_5716_MOESM1_ESM.pdf]

Epithelial-derived IL-33 promotes intestinal tumorigenesis in *Apc*$^{Min/+}$ mice

Zhengxiang He[1, †], Lili Chen[1, †], Fabricio O. Souto [1], Claudia Canasto-Chibuque[1], Gerold Bongers[1], Madhura Deshpande[1], Noam Harpaz[2], Huaibin M. Ko[2], Kevin Kelley[3], Glaucia C. Furtado[1] & Sergio A. Lira[1*]

[1] Precision Immunology Institute, Icahn School of Medicine at Mount Sinai, New York, 10029, USA

[2] Department of Pathology, Icahn School of Medicine at Mount Sinai, New York, 10029, USA

[3] Department of Developmental and Regenerative Biology, Icahn School of Medicine at Mount Sinai, New York, 10029, USA

[†] These authors contributed equally to this work.

* **Address correspondence to:** Sergio A. Lira, M.D. Ph.D., 1425 Madison Ave, Box 1630 Room 12-20, New York, NY 10029. Phone (212) 659-9404; FAX (212) 849-2525; E-mail: sergio.lira@mssm.edu.

Supplementary Figures

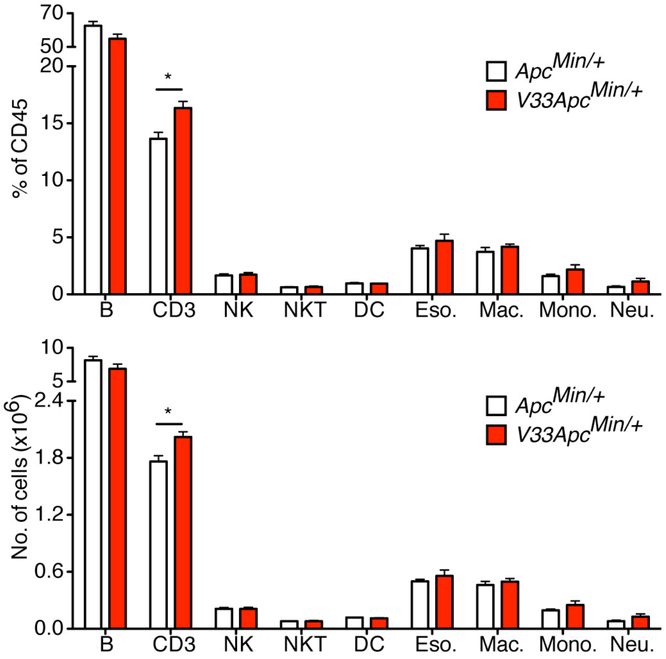

Fig. S1. Analysis of cellularity in the colon of *Apc*$^{Min/+}$ mice and *V33 Apc*$^{Min/+}$ mice.

Relative and absolute number of B cells (B220^{+}), T cells (CD3^{+}), NK (NK1.1^{+}), NKT (NK1.1^{+}CD3^{+}), DC (CD11c^{+}F4/80^{-}MHC-II^{+}), eosinophils (CD11b^{+}siglecF^{+}), macrophages (CD11b^{+}Ly6c^{-}Ly6G^{-}F4/80^{hi}), monocytes (CD11b^{+}Ly6c^{+}), neutrophils (CD11b^{+}Ly6G^{+}) in the colon of *Apc*$^{Min/+}$ mice and *V33Apc*$^{Min/+}$ mice at d120. Graphs show means ± s.e.m..*P < 0.05, nonparametric Mann-Whitney test.

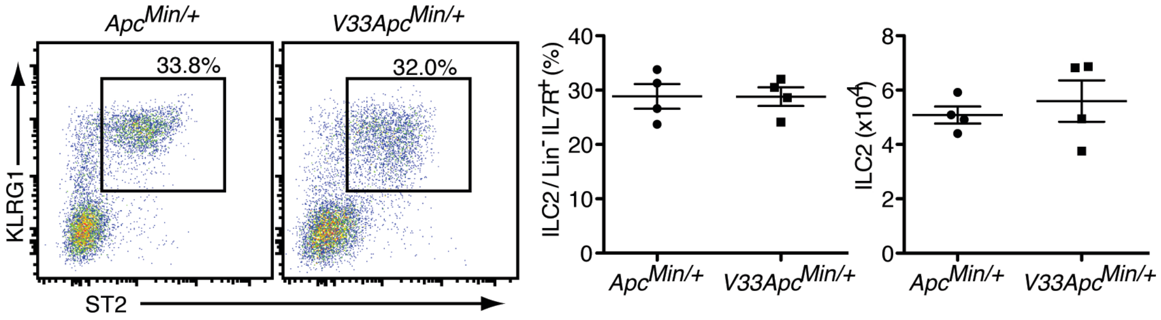

Fig. S2. Analysis of ILC2s in the colon of *V33 Apc*$^{Min/+}$ mice.

Relative and absolute number of ILC2s ($CD45^{+}Lin^{-}IL7R^{+}KLRG1^{+}ST2^{+}$) in the colon of *Apc*$^{Min/+}$ mice and V33 *Apc*$^{Min/+}$ mice at d120. Left, representative flow cytometry plots gated on $CD45^{+}$ cells; right, statistical data show means ± s.e.m..

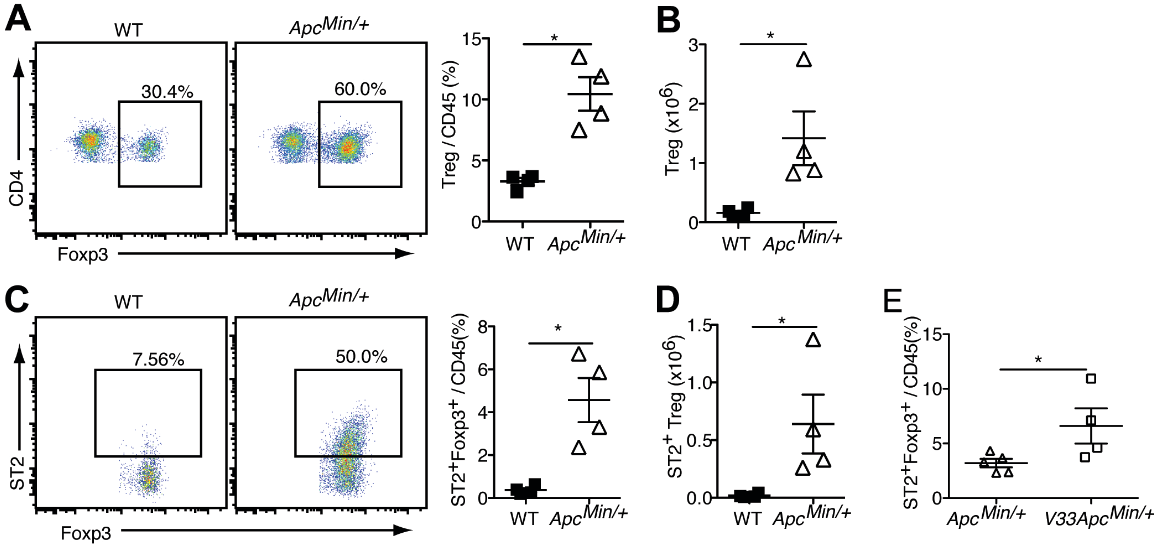

Fig. S3. Epithelial-derived IL-33 signaling expands ST2$^+$ Treg cells in the small intestine.

(**a**) Relative number of Treg cells (CD4$^+$Foxp3$^+$) in the small intestine of *Apc*$^{Min/+}$ mice and WT mice at d120. Left, representative flow cytometry plots gated on CD45$^+$ cells; right, statistical data show means ± s.e.m.. (**b**) Absolute number of Treg cells (CD4$^+$Foxp3$^+$) in the small intestine of *Apc*$^{Min/+}$ mice and WT mice at d120. (**c**) Relative number of ST2$^+$Treg cells in the small intestine of *Apc*$^{Min/+}$ mice and WT mice at d120. Left, representative flow cytometry plots gated on CD45$^+$ CD4$^+$ cells; right, statistical data show means ± s.e.m.. (**d**) Absolute number of ST2$^+$Treg cells in the small intestine of *Apc*$^{Min/+}$ mice and WT mice at d120. (**e**) Relative number of ST2$^+$Treg cells in the small intestine of *Apc*$^{Min/+}$ mice and *V33 Apc*$^{Min/+}$ mice at d120. Graphs show means ± s.e.m..*P < 0.05, nonparametric Mann-Whitney test.

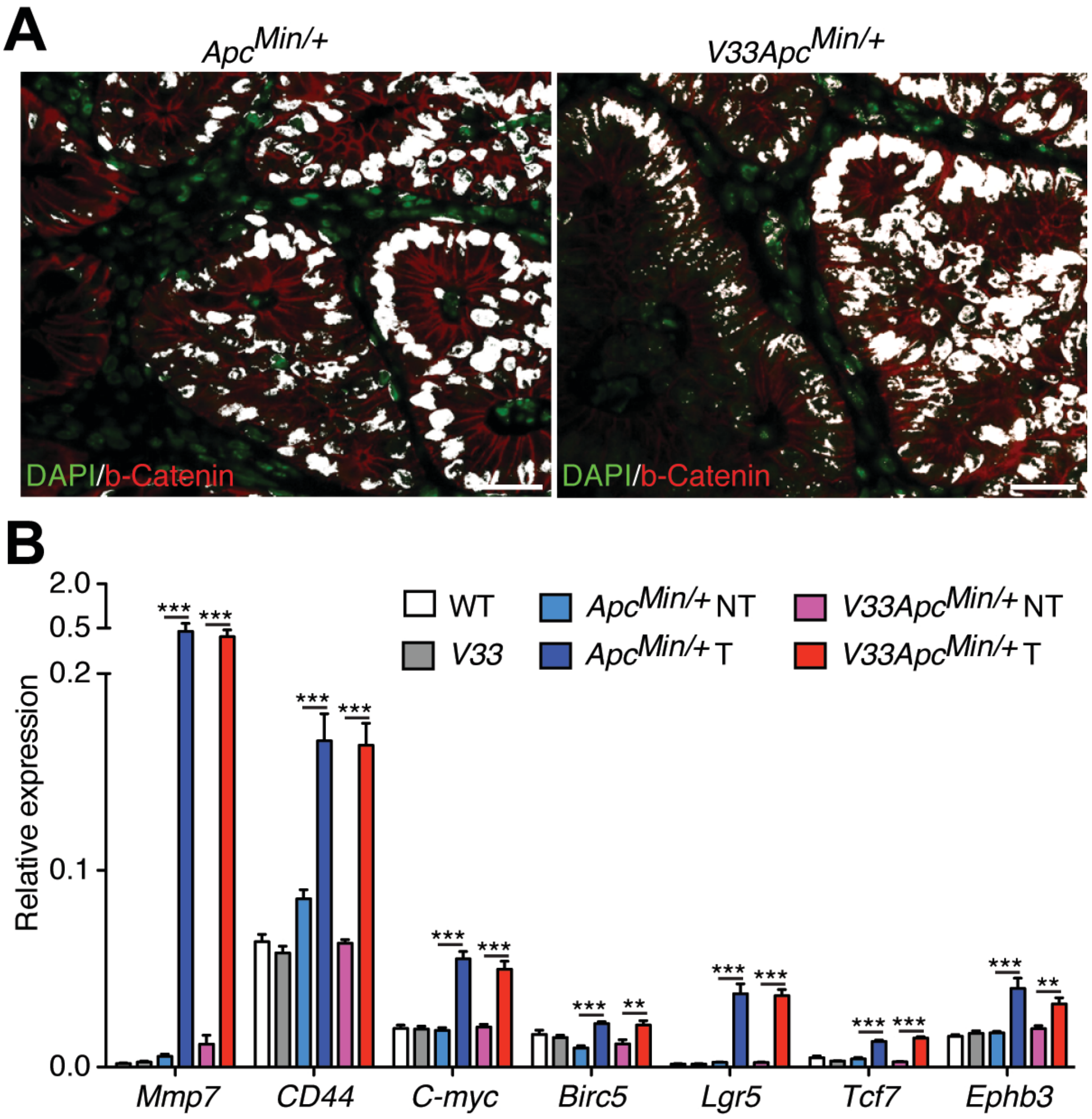

Fig S4. IL-33 promotes intestinal tumorigenesis independent of the β-catenin pathway in IEC.

(**a**) Representative β-catenin staining of polyps of *Apc*$^{Min/+}$ mice and *V33 Apc*$^{Min/+}$ mice at d120. Pictures stained with DAPI (green) and cytoplasmic β-catenin (red) are shown. Nuclear translocation (double staining) is indicated in white. Scale bars, 50μm. (**b**) Relative expression levels of β-catenin pathway target genes were analyzed by qPCR in colon of size-matched tumor area (T) and adjacent

non-tumor normal area (NT) of *Apc*$^{Min/+}$ mice and *V33 Apc*$^{Min/+}$ mice at d120. Data were normalized to the expression levels of the Ubiquitin transcript. Graphs show means ± s.e.m.. ** $P < 0.01$, ***$P < 0.001$, one-way ANOVA.

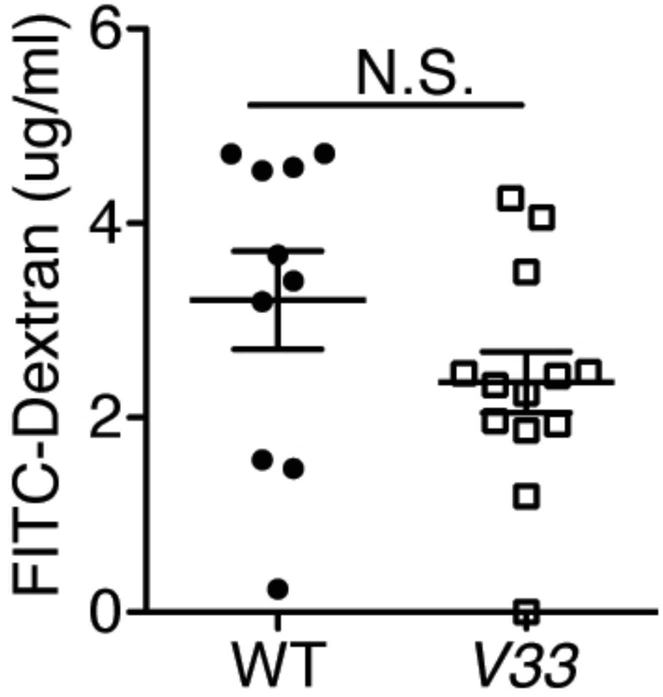

Fig. S5. Intestinal permeability in *V33* mice.

Intestinal permeability in *V33* and WT mice assessed by measuring serum FITC-Dextran levels 5 h after administration. Graphs show means ± s.e.m., N.S. means not significant.

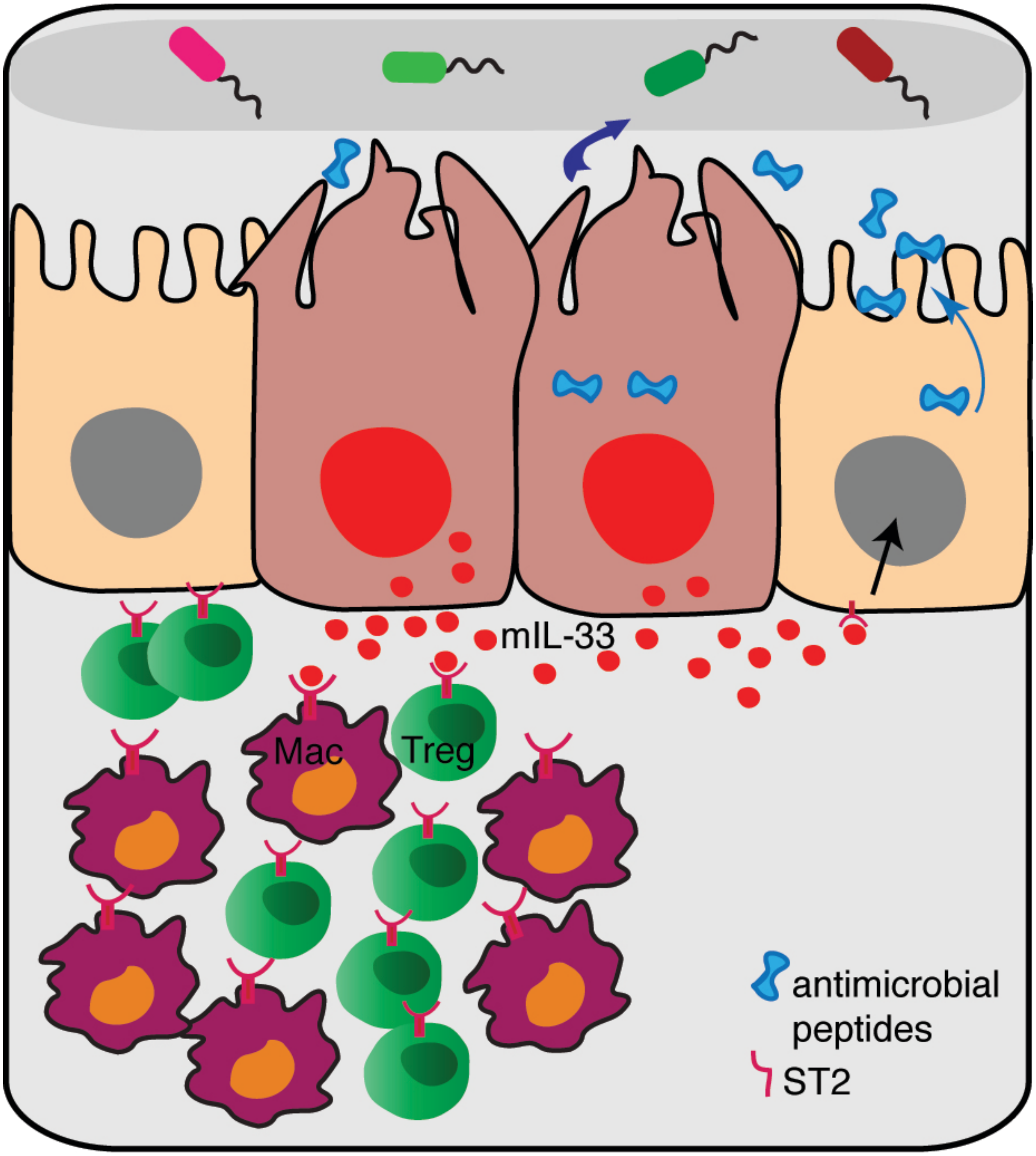

Fig. S6. A model for the role of epithelial-derived IL-33 in the intestinal tumorigenesis in *Apc* $^{Min/+}$ mice.

IL-33 is expressed by epithelial cell in adenomatous areas in *Apc*$^{Min/+}$ mice. Increased expression of IL-33 in epithelium induces a pro-tumor environment by

expanding the number of $ST2^+$ Tregs and induction of alternatively activated macrophages. Epithelial-derived IL-33 alters expression of antimicrobial genes, promoting dysbiosis and favoring colonic tumor development.
